# Factors Associated with Unmet Healthcare Needs in Serbia Before and During the COVID-19 Pandemic: Analysis Based on EU-SILC Data

**DOI:** 10.3390/healthcare14050599

**Published:** 2026-02-27

**Authors:** Milena Jakovljevic, Bojana Matejic, Milena Santric-Milicevic, Zeljka Stamenkovic, Ivana Sotirovic, Miodrag Milenovic, Verica Todorov-Sakic, Andja Cirkovic

**Affiliations:** 1 Department for Analysis, Planning and Organization of Health Care, City Institute of Public Health Belgrade, 11000 Belgrade, Serbia; 2 Institute of Social Medicine, Faculty of Medicine, University of Belgrade, 11000 Belgrade, Serbia; bojana.matejic@med.bg.ac.rs (B.M.); milena.santric-milicevic@med.bg.ac.rs (M.S.-M.); znikolic21@gmail.com (Z.S.); sotirovic.ivana@gmail.com (I.S.); 3 Department of Anesthesiology and Intensive Care, Emergency Center, University Clinical Center of Serbia, 11000 Belgrade, Serbia; milenx67@gmail.com; 4 Department of Surgery and Anesthesiology, Faculty of Medicine, University of Belgrade, 11000 Belgrade, Serbia; 5 Clinical Department for Nephrology, University Medical Center Zvezdara, 11000 Belgrade, Serbia; todorovverica@yahoo.com; 6 Institute for Medical Statistics and Informatics, Faculty of Medicine, University of Belgrade, 11000 Belgrade, Serbia; andjacirkovic01@gmail.com

**Keywords:** unmet healthcare needs, COVID-19, inequities

## Abstract

**Background/Objectives:** The COVID-19 pandemic substantially affected both the provision and demand for health services. There are few studies that analyzed factors associated with unmet healthcare needs during the COVID-19 pandemic relative to regular pre-pandemic period, mainly in high-income countries. This study examines the change in the likelihood of reporting unmet healthcare needs as well as individual demographic, socioeconomic, health-related, and geographical characteristics associated with unmet healthcare needs during the COVID-19 pandemic and during the pre-pandemic period in Serbia. **Methods:** We utilized data from the Survey on Income and Living Conditions in the Republic of Serbia for 2019 and 2021. Multivariable logistic regression analysis was conducted on the pooled sample comprising 21,422 respondents aged 16 years and older from both survey years. **Results:** Overall, respondents had 1.6 times higher odds of reporting unmet healthcare needs during the COVID-19 pandemic compared with the pre-pandemic period. In the pooled multivariable analysis, older adults (OR = 2.32), individuals reporting poor or very poor self-rated health (OR = 2.16), and those with chronic diseases (OR = 1.46) were more likely to report unmet healthcare needs. In contrast, individuals with higher levels of education (ORs = 0.72 for high school, 0.65 for college, and 0.51 for master’s degree), retired individuals (OR = 0.70), inactive persons (OR = 0.76), and students/pupils (OR = 0.22) had significantly lower odds of unmet needs, compared to their counterparts. Relative to the poorest income quintile, individuals in all higher income quintiles were less likely to report unmet healthcare needs, with the lowest odds observed in the 5th quintile (OR = 0.49). Residents of thinly populated areas had higher odds compared to those in densely populated areas (OR = 1.19). **Conclusions:** Identified associations with unmet healthcare needs should be used to develop targeted strategies for strengthening healthcare access, particularly in the context of future crises.

## 1. Introduction

During the COVID-19 pandemic, many patients encountered unmet healthcare needs that were significantly fueled by strict anti-epidemic measures and overburdened health systems [1,2,3,4,5]. The post-COVID era reports on mortality and morbidity insufficiently discuss what happened to the patients who struggled to access essential medical services and obtain the necessary support for chronic conditions, prevention, screening, or non-emergency treatments during the prolonged pandemic. According to Organization for Economic Cooperation and Development (OECD) data for 2020, the number of in-person consultations, hospital stays for cancer care, and elective surgeries in European Union (EU) countries decreased by 17%, 11.5%, and 14%, respectively. Hospital admissions for acute myocardial infarction and cerebrovascular disease declined, indicating a compromise in timely access to acute care [3]. In Serbia, the use of primary healthcare by the adult population dropped by 15% in 2020 compared to the previous year [6]. Additionally, outpatient hospital visits, admissions, and day procedures significantly decreased [7]. On the other hand, the pervasive fear of contracting the virus contributed to a notable decline in the demand for healthcare services [8,9].

Measuring and reporting unmet healthcare needs, together with identifying the most vulnerable population groups during a crisis, underscores the importance of equity considerations in health system performance [10,11]. Self-reported unmet healthcare needs assessment remained the most widely used measurement method during the COVID-19 pandemic, as its validity has been demonstrated through strong associations with worsening health outcomes [12,13,14,15]. According to Eurofound’s Living, working, and COVID-19 e-survey, more than one in five people across EU countries reported forgoing medical care and nearly one in five did so as late as spring 2021 [16]. This trend aligns with findings from 39 low-income or middle-income countries in 2020, which indicate that one-fifth of households did not receive the necessary healthcare due to fear of infection, movement restrictions, or financial difficulties [17].

Assessing unmet healthcare needs and identifying their predictors during crises provides an important equity perspective for healthcare policy development in the post-crisis period. Experts agree that the current efforts in monitoring universal health coverage fail to reflect the extent of unmet needs nationally and globally [10]. Nonetheless, self-reporting of unmet healthcare needs is highly relevant for localizing the interventions [18].

Few studies that explore how predictors of unmet healthcare needs have changed during the COVID-19 pandemic, compared to pre-pandemic circumstances, indicate that lower income levels and poorer general health are associated with unmet healthcare needs in both periods. Additionally, those suffering from chronic illness become particularly vulnerable during the pandemic, and the impact of predisposing factors varies across surveys [19,20]. Utilizing Andersen’s Behavioral Model of Health Services Use as a theoretical framework provides a valuable approach to understanding and addressing unmet healthcare needs. This model categorizes key predictors into three main components, each of which can be modified to varying extents: predisposing factors (e.g., gender, age, education, marital status, and employment status), enabling factors (e.g., income), and need factors (e.g., self-perceived general health and the presence of chronic illnesses), [18,21].

In countries profoundly impacted by COVID-19 mortality, especially where it was the leading cause of death as with Serbia [22], disclosing the changes in unmet healthcare needs is a moral and professional obligation. To fill in the actionable information gap in order to ensure equitable access to healthcare, this study explores the frequency and predictors of unmet healthcare needs in Serbia’s population aged 16 and older before and during the COVID-19 pandemic, using Andersen’s Model. The study assess change in the odds of reporting unmet healthcare needs in 2021 compared with 2019.

### A Brief Overview of the Healthcare System of the Republic of Serbia

The Serbian healthcare system is primarily financed through compulsory health insurance contributions, which amount to 10.3% of payroll taxes, with the state budget covering healthcare for vulnerable population groups. The system formally guarantees access to a comprehensive package of healthcare services for nearly the entire population, with mandatory health insurance covering approximately 98% of residents. However, financial protection is limited by co-payments, particularly for outpatient medicines and medical products [23].

Public health expenditure is relatively high, accounting for 9.7% of GDP, but out-of-pocket payments remain substantial, representing 32.4% of total health expenditure. Although OOP payments have declined since 2017, they are still substantially higher than the EU average [24].

The majority of healthcare services in Serbia are delivered through an extensive network of state-owned providers. Healthcare is organized across three interconnected levels—primary, secondary, and tertiary care. Primary care is provided by a “chosen doctor”, with patients assigned to a primary healthcare center in their area of residence. Secondary care includes both outpatient and inpatient hospital services. In addition, a burgeoning private healthcare sector operates alongside the public system [23]. Republic Health Insurance Fund contracts private providers only for a very limited number of services [25].

## 2. Materials and Methods

### 2.1. Study Design, Participants, and Data Source

This study is a secondary analysis of data collected in the Survey on Income and Living Conditions (SILC) in the Republic of Serbia, excluding Kosovo and Metohija, in 2019 and 2021. The SILC is a nationally representative survey conducted every year in European countries. It provides comparable cross-sectional and longitudinal data on income, poverty, social exclusion, and living conditions.

A two-stage stratified sample is used for conducting SILC in Serbia. The observation units are private households and current household members aged 16 and above. The 2011 Census of Population, Households, and Dwellings in the Republic of Serbia is used as the sampling framework. Census circles, stratified by settlement type (densely populated, intermediate, and thinly populated areas), as well as Nomenclature of territorial units for statistics 2 (NUTS2)-level regions (Belgrade Region, Region of Vojvodina, Region of Sumadija and Western Serbia, and the Region of Eastern and Southern Serbia), comprise the first-stage units. The second-stage units comprise households and their members.

The 2019 survey was conducted from May to August 2019, and the 2021 survey from May to July 2021. A team of face-to-face interviewers collected data in the field using computer-assisted personal interviewing (CAPI). A household questionnaire and an individual questionnaire were used [26,27]. The household questionnaire, in addition to the household information panel and a list of household members, contains questions on the demographic characteristics of all members, taking care of children, housing and housing costs, quality of life, household income, and agricultural activity. The personal questionnaire covers demographic characteristics, education, economic activity, income, and individual quality of life. The head of the household answered household questions, while personal questions were answered directly by each household member aged 16 and above. A total of 5130 households and 13,733 persons were surveyed in 2019, and 5158 households and 13,855 persons in 2021.

We used SILC datasets from 2019 and 2021 because they provide results for the one year before and one year during the COVID-19 pandemic, respectively. As SILC examines unmet healthcare needs over the previous 12 months, this analysis excludes the 2020 dataset, which spans the period before and after the COVID-19 outbreak. The datasets used did not contain any missing data.

To ensure independence of observations in this study, we excluded from the 2021 dataset all respondents who had already participated in the 2019, identified by matching unique personal identification numbers. This step was necessary because SILC has a rotating panel design, in which subsamples participate for four consecutive years, resulting in partial overlap of individuals across survey waves. The final analytical samples therefore comprised 13,733 respondents in 2019 and 7689 respondents in 2021.

### 2.2. Variables

The dependent variable in this survey refers to self-reported unmet needs for physician services, based on the question: “Was there any time during the past 12 months that you should have visited a doctor but did not? Yes/No”. A “yes” answer indicates that the participant perceived unmet healthcare need during the past 12 months.

Applying Andersen’s model of healthcare utilization to select and categorize independent variables, we divided them into predisposing, enabling/disabling, and need factors, while also incorporating geographical determinant. Predisposing factors include sex, age groups (16–29; 30–49; 50–64; 65+), marital status (married; never married; divorced/separated; widowed), education (elementary or less; high school; college/university; master/doctorate), and self-defined current employment status (employed; unemployed; retired; other inactive person; student/pupil). Although there were differences in the suggested responses regarding education level and employment status between the 2019 and 2021 questionnaires, it was possible to align these responses using the SILC methodologies [26,27]. Response categories available in 2021 (upper secondary education general and upper secondary education vocational) were merged into a single category (upper secondary education), which was the option in 2019. Regarding employment status, response categories from 2019 were merged to align with the category used in 2021 (employee working full time, employee working part time, self-employed working full time, self-employed working part time merged into the emloyed category). Enabling factors include household income (quintiles) and degree of urbanization (densely populated; intermediate populated; thinly populated areas). Quintiles are formed based on equivalized household income, representing the total disposable household income evenly distributed among members according to the modified OECD equivalence scale. This scale attributes a weight to all members of the household: 1.0 to the first adult, 0.5 to the second and each subsequent person aged 14 and above, and 0.3 to each child aged below 14. Needs for healthcare were assessed through three questions on health status: self-perceived general health (very good/good; fair; bad/very bad), chronic illness (yes; no), limitation in activities because of long-standing health problems (very limited; limited; not limited). We used the region of residence (Serbia-North; Serbia-South) as a geographical determinant. Due to SILC disclosure control rules, the geographical determinant was limited to NUTS1-level regions, Serbia-North, and Serbia-South, rather than NUTS2-level regions [28].

To assess the impact of the COVID-19 pandemic on the likelihood of reporting unmet healthcare needs compared with the pre-pandemic period, the study year (2019; 2021), was included as an independent variable in the analysis.

### 2.3. Statistical Analysis

Descriptive statistical analysis (absolute numbers and percentages) was performed to examine the characteristics of the survey population and the frequency of unmet healthcare needs in 2019 and 2021. Pearson’s chi-squared test was used to test differences in the frequencies of unmet healthcare needs between 2019 and 2021 across examined characteristics. To identify factors associated with unmet healthcare needs and to assess change in the odds of reporting unmet healthcare needs in 2021 compared with 2019, multivariable logistic regression analysis was performed on the pooled sample, including respondents from both study years. Independent variables, including survey year, were entered into multivariable logistic regression analysis to estimate the adjusted difference in unmet healthcare needs between 2021 and 2019 and the adjusted associations of predictors with unmet healthcare needs across both study periods. Results of logistic regression analysis are presented as odds ratios (ORs) with corresponding 95% confidence intervals.

All analyses were conducted using the Statistical Package for the Social Sciences software (SPSS 25.0 for Windows, SPSS Inc., IBM, Armonk, NY, USA). A *p*-value of less than 0.05 was considered significant. Multicollinearity was assessed using variance inflation factors (VIFs).

## 3. Results

The analysis included citizens of Serbia aged 16 years and older, comprising 13,733 respondents in 2019 and 7689 respondents in 2021. Regarding the characteristics of the study populations, no statistically significant differences between 2019 and 2021 were observed in sex distribution, degree of urbanization, or regional distribution. In both study populations, women slightly predominated (around 51% in both years), most respondents lived in thinly populated areas (almost 45%), and a higher proportion of respondents resided in Southern Serbia (58%).

Statistically significant differences between 2019 and 2021 were observed in age structure, marital status, educational attainment, employment status, income quintiles, self-perceived general health, the presence of chronic disease, and limitations in activities due to health problems. Compared with 2019, in 2021, the share of individuals aged 65 years and older was higher (27.7% vs. 29.6%), the proportion of respondents with lower education was lower (elementary school or less: 29.7% vs. 27.8%), more respondents perceived own health as very good or good (57.5% vs. 62.8%), and more respondents had no limitations due to health problems (85.4% vs. 87.4%).The share of chronically ill patients was higher in 2021 (32.9% vs. 34.9%) (Table 1).

Around 10% of participants reported unmet healthcare needs in 2019, with a significantly higher value of 13.8% in 2021. The frequency of unmet healthcare needs was significantly higher in 2021 compared with 2019 among both sexes, in age groups 50–64 and 65+, among married, separated/divorced, and widowed individuals, across all education levels, among retired and inactive individuals, in all income quintiles except the poorest, across all degrees of urbanization, and in both regions. Regarding health-need factors, the frequency of unmet needs was higher in 2021 among individuals reporting fair or poor/very poor health and among those with chronic disease. Among the youngest age group and employed individuals, frequency of unmet needs was lower in 2021 compared with 2019 (Table 2).

Across the population of 16+ years old in Serbia, the odds of reporting unmet healthcare needs in 2021 were 1.6 times higher compared with 2019. According to the results of multivariable analysis conducted on the pooled sample, three predisposing factors—age, education, and employment status—were significant for unmet healthcare needs across both years. Compared to individuals aged 16–29 years, those aged 30–49 (OR = 1.80), 50–64 (OR = 2.30), and 65+ (OR = 2.32) had significantly higher odds of reporting unmet needs. Those with high school (OR = 0.72), college/university (OR = 0.65), and master’s/doctoral education (OR = 0.51) had reduced odds compared to individuals with elementary education or less. Regarding employment status, retired (OR = 0.70), inactive (OR = 0.76), and students/pupils (OR = 0.22) were significantly less likely to report unmet healthcare needs (Table 3).

A clear income gradient was observed. Compared to the poorest income quintile, individuals in all higher-income groups had lower odds of unmet healthcare needs, with the lowest odds among the 5th quintile (OR = 0.49). Residents of thinly populated areas were more likely to report unmet needs compared to those in densely populated areas (OR = 1.19). Regarding health-need factors, relative to individuals reporting very good/good health, those reporting fair (OR = 1.85) and bad/very bad health (OR = 2.16) had significantly higher odds, as well as those with chronic disease (OR = 1.46). In the pooled multivariable analysis, sex and region of residence were not significantly associated with unmet healthcare needs (Table 3).

Detailed results of the multivariate logistic regression analyses for 2019 and 2021 separately, with all factors included and corresponding estimates, are presented in the Supplementary Material. In both 2019 and 2021, higher odds of unmet healthcare needs were associated with older age, status of separated or divorced person, lower income quintiles, living in thinly populated areas, poorer self-rated health, and activity limitations due to health problems. In 2019, lower odds of unmet healthcare needs were additionally observed among women and individuals with higher educational attainment, while employment status showed a protective effect among students, retirees, and inactive individuals. In 2021, only the student status had a protective effect, while the presence of chronic disease was a significant factor and increased the odds for unmet needs. Sex, education, and region of residence were not significantly associated with unmet healthcare needs in 2021 (Table S1).

## 4. Discussion

As far as we know, this was the first study that examined the frequency, likelihood, and predictors of unmet healthcare needs during the COVID-19 pandemic and during the pre-pandemic period in Serbia. According to our findings, the frequency of unmet healthcare needs increased from 10.2% in 2019 to 13.8% in 2021. Overall, citizens of Serbia aged 16 or more were 1.6 times more likely to report unmet healthcare needs during the COVID-19 pandemic compared to the pre-pandemic period. These findings align with other studies reporting substantial increases in unmet healthcare needs during the pandemic [19,20,29,30,31]. COVID-related circumstances, including the overburdening of healthcare human and spatial capacities with COVID-19 patients on the supply side, as well as anti-epidemic measures, including avoiding social contact, and the fear of contracting the virus on the demand side, reduced healthcare accessibility and increased healthcare avoidance, thereby contributing to the rise in unmet healthcare needs.

Considering the negative consequences of COVID-19, the observed differences in sample composition between 2021 and 2019 regarding age and self-rated health require additional interpretation. First, the higher proportion of individuals aged 65 years and older in 2021 compared with 2019 may be attributed to pronounced demographic aging trends in Serbia [32], which appear to have prevailed over the effects of increased COVID-19-related mortality among the elderly. Second, the higher share of respondents reporting good self-rated health during the pandemic may reflect a change in subjective health evaluation across the population rather than realistic improvements, with uninfected individuals perceiving their health more positively during crisis [33].

Although sex was not a significant predictor of unmet healthcare needs when both study periods were analyzed together, stratified analysis of the pre-pandemic period indicated that women had slightly lower odds of unmet healthcare needs compared to men. Evidence from other countries suggests that women were at an even higher risk for unmet healthcare needs during the pandemic [19,29,34]. Increased caregiving responsibilities during COVID-19—as women disproportionately assumed caregiving and domestic duties—may have led them to postpone or forego seeking care for their own health needs [16]. In addition, widespread disruptions to reproductive and preventive healthcare services during the pandemic [3,35] may have disproportionately affected women, thereby contributing to their unmet needs, especially considering their higher utilization of these services in non-crisis settings [36].

Older age emerged as an independent predictor of unmet healthcare needs, while some previous studies did not identify such an association during the COVID-19 pandemic [19,20,30,34,37]. These discrepancies may partly reflect less restrictive anti-epidemic measures, including movement restrictions and social distancing measures, applied to older populations in other settings [19]. Secondly, well-established prioritization policies for the most vulnerable populations (particularly older adults) likely protected their access to essential care during the pandemic, whereas younger individuals more frequently experienced interruptions in preventive and elective services [30,37]. Despite the existence of strategic and operational documents aimed at improving healthcare access for older adults in Serbia [38,39], there remains a pressing need to address their unmet healthcare needs both during public health crises and under regular conditions.

Higher educational attainment decreased the likelihood of unmet healthcare needs, consistent with previous Serbian studies [40,41]. However, in the stratified analysis of the pandemic period protective effect of higher education disappeared and level of education was not among significant predictors of unmet healthcare needs. Greater health literacy [42], better self-awareness of personal health, and better access to healthcare in regular circumstances [43], translating into better ability to recognize and fulfill healthcare need, may have made more educated individuals more sensitive to pandemic-related access limitations. More frequent use of health services among these groups under regular conditions [44] supports this argument as well. The heightened sensitivity probably led them to perceive and report unmet needs more readily during the pandemic, thereby vanishing [19] or even reversing [34] the usual education-related protective effect.

The protective effect of non-employment status (retired and inactive individuals), observed when both study periods were considered together, was not present during the pandemic separately. This finding is consistent with previous research showing that inactive and retired individuals were more exposed to pandemic-related unmet healthcare needs, including postponed treatments, providers’ closure, and avoidance of care due to fear of infection [29]. Pandemic-related movement restrictions and social distancing measures appear to have disproportionately affected non-employed groups, increasing their social isolation [45] and potentially making it more difficult for them to access healthcare services amid the disruptions caused by the COVID-19 pandemic. Mentioned anti-epidemic measures in Serbia were implemented with particular emphasis on the pensioner population, making them even more exposed to the effects of isolation [46].

Economic status was an important enabling factor for unmet healthcare needs; lower income was consistently associated with higher unmet needs, in line with previous research [19,40], but the corresponding disparities became narrower in the pandemic period analyzed separately. This convergence should not be viewed as a reduction in inequality. Rather, it probably reflects widespread restrictions in access to healthcare and the avoidance of health services due to fear of infection, affecting individuals regardless of their ability to pay out-of-pocket or use private healthcare services. This interpretation is supported by another study indicating the absence of a significant association between income and pandemic-related unmet healthcare needs [29], as well as by findings indicating the absence of income-related horizontal inequity in unmet needs across countries with substantially different healthcare systems and levels of health expenditure [47].

Individuals residing in densely populated areas experienced a lower likelihood of unmet healthcare needs than those living in intermediate areas, when both study periods were considered together. This disparity disappeared when the pandemic period was analyzed separately. Higher COVID-19 transmission rates in densely populated areas [48,49] led to healthcare system overload due to the increased number of COVID-19 patients, thereby reducing the availability of non-COVID services and contributing to a disproportionate rise in unmet healthcare needs in urban centers. On other hand, compared with residents of thinly populated areas, individuals living in densely populated areas are in a more favorable position, reporting less unmet needs. Considering the fact that the most significant healthcare access challenges in Serbia are in thinly populated areas [50], which is consistent with international evidence indicating lower access and higher levels of unmet needs in rural settings [51,52], the disparity between densely and thinly populated areas is not unexpected.

The presence of chronic conditions has been identified as an important determinant of unmet healthcare needs across diverse healthcare settings during COVID-19 [19,20,34], suggesting that this vulnerability was pandemic-driven rather than context-specific. These evidences reflects reduced physician follow-ups and disruptions in the continuity of care for chronically ill patients during the pandemic [3,53]. The findings related to health-need factors, particularly the presence of chronic conditions, underscore concerns about the deterioration of health among those already facing challenges and highlight deepening vulnerabilities due to unmet healthcare needs during crises. To improve preparedness for future crises, it is crucial to establish strong and precise prioritization criteria within health systems, as well as to assess and provide the necessary healthcare resources to protect the most vulnerable populations.

### 4.1. Study Limitations

This study has several limitations. First, the self-reported nature of the information collected may lead to recall bias. Self-reported unmet healthcare needs reflect individuals’ subjective perceptions and expectations of the healthcare system but do not capture unrecognized clinically relevant needs. Second, the SILC addresses unmet needs for physician services in general, making it difficult to distinguish between different levels of healthcare needed (e.g., primary versus secondary care). There is also a lack of data on other health services, such as laboratory or diagnostic services. Third, although the use of nationally representative samples is a strength of the study, a limitation regarding the differences observed in the samples structure between the two study periods should be noted.

### 4.2. Policy and Practice Implications

Decision-makers should leverage evidence on unmet healthcare needs under regular pre-pandemic and pandemic conditions. Targeted outreach, flexible service delivery models, and prioritization policies may help mitigate access barriers especially during future public health crisis. The elevated risk observed among the elderly and individuals with chronic illnesses highlights the need to prioritize continuity of long-term and chronic disease management. Expanding home- and community-based services, as well as outsourcing certain services to the private sector, could help maintain access for these groups. It is also important to address organizational and informational barriers that may disproportionately affect economically inactive populations during crises. Finally, to mitigate challenges in thinly populated areas, specific resource allocation and locally adapted solutions are essential for strengthening healthcare access, particularly in the context of future crises.

## 5. Conclusions

Our study found that the odds of reporting unmet healthcare needs among individuals aged 16 and older in Serbia were 1.6 times higher during the COVID-19 pandemic compared to the pre-pandemic period, The groups adversely affected included older adults, those with persistent health needs, as well as residents of thinly populated areas. Identified associations with unmet healthcare needs should be used to develop targeted strategies for strengthening healthcare access, particularly in the context of future crises.

## Figures and Tables

**Table 1:** Characteristics of the study population, before and during the COVID-19 pandemic.

Variable	Category	Before COVID-19 (2019)		During COVID-19 (2021)		*p* *
		*n*	%	*n*	%	
Sex	Male	6656	48.5	3747	48.7	0.360
	Female	7077	51.5	3942	51.3	
Age group (years)	16–29	2368	17.2	1411	18.4	<0.001
	30–49	4065	29.6	2071	26.9	
	50–64	3491	25.4	1933	25.1	
	65+	3809	27.7	2274	29.6	
Marital status	Married	7796	56.8	4282	55.7	0.001
	Never married	3454	25.2	2090	27.2	
	Separated and divorced	728	5.3	345	4.5	
	Widowed	1755	12.8	972	12.6	
Education level ^a^	Elementary or less	4083	29.7	2135	27.8	<0.001
	High school	7446	54.2	4162	54.1	
	College/university	1973	14.4	1275	16.6	
	Master/doctorate	231	1.7	117	1.5	
Employment status	Employed	4939	36.0	2810	36.5	<0.001
	Unemployed	2879	21.0	1282	16.7	
	Retired	4154	30.2	2364	30.7	
	Inactive ^b^	829	6.0	517	6.7	
	Student, pupil	932	6.8	716	9.3	
Income quintile	1—poorest	2888	21.0	1578	20.5	0.027
	2	2976	21.7	1580	20.5	
	3	2867	20.9	1560	20.3	
	4	2548	18.6	1503	19.5	
	5—richest	2454	17.9	1468	19.1	
Degree of urbanization ^c^	Densely populated area	3956	28.8	2278	29.6	0.135
	Intermediate area	3598	26.2	2060	26.8	
	Thinly populated area	6179	45.0	3351	43.6	
Self-perceived general health	Very good/good	7895	57.5	4828	62.8	<0.001
	Fair (neither good nor bad)	3573	26.0	1881	24.5	
	Bad/very bad	2265	16.5	980	12.7	
Chronic disease	No	9221	67.1	5003	65.1	0.001
	Yes	4512	32.9	2686	34.9	
Limitation in activities due to health problems	Not limited	11726	85.4	6723	87.4	<0.001
	Limited	1348	9.8	654	8.5	
	Strongly limited	659	4.8	312	4.1	
Region ^d^	Serbia-North	5721	41.7	3226	42.0	0.341
	Serbia-South	8012	58.3	4463	58.0	

* Pearson’s chi-squared test; ^a^ ISCED levels grouped in accordance with education system in Serbia: elementary school or less = ISCED 2 or below, high school = ISCED 3, college/universaty = ISCED 4–6, master/doctorate = ISCED 7–8; ^b^ inactive refers to being unable to work due to long-standing health problems or fulfilling domestic tasks or other duties; ^c^ division according to DEGURBA classification: densely populated area—contiguous grid cells of 1 km^2^ with a density of at least 1500 inhabitants per km^2^ and a minimum population of 50,000, intermediate area—clusters of contiguous grid cells of 1 km^2^ with a density of at least 300 inhabitants per km^2^ and a minimum population of 5000, thinly populated area—grid cells outside urban clusters; ^d^ NUTS1-level region Serbia-North includes NUTS2-level regions: Belgrade Region and Region of Vojvodina; NUTS1 level-region Serbia-South includes NUTS2-level regions: Region of Sumadija and Western Serbia and Region of Eastern and Southern Serbia.

**Table 2:** Frequency of unmet healthcare needs before and during the COVID-19 pandemic, by examined characteristics.

Variable	Category	Before COVID-19 (2019)		During COVID-19 (2021)		*p* *
		*n*	%	*n*	%	
Sex	Male	680	10.2	604	15.3	<0.001
	Female	722	10.2	459	12.2	<0.001
Age group (years)	16–29	82	3.5	29	2.1	0.008
	30–49	347	8.5	158	7.6	0.120
	50–64	464	13.3	304	15.7	0.008
	65+	509	13.4	572	25.2	<0.001
Marital status	Married	802	10.3	598	14.0	<0.001
	Never married	211	6.1	127	6.1	0.505
	Separated/divorced	106	14.6	70	20.3	0.012
	Widowed	283	16.1	268	27.6	<0.001
Education level ^a^	Elementary or less	698	17.1	417	19.5	0.010
	High school	592	8.0	503	12.1	<0.001
	College/ university	106	5.4	134	10.5	<0.001
	Master/doctorate	6	2.6	9	7.7	0.030
Employment status	Employed	407	8.2	197	7.0	<0.028
	Unemployed	370	12.9	154	12.0	0.242
	Retired	492	11.8	585	24.7	<0.001
	Inactive ^b^	123	14.8	119	23.0	<0.001
	Student, pupil	10	1.1	8	1.1	0.557
Income quintile	1—poorest	501	17.3	289	18.3	0.221
	2	340	11.4	268	17.0	<0.001
	3	243	8.5	197	12.6	<0.001
	4	188	7.4	171	11.4	<0.001
	5—richest	130	5.3	138	9.4	<0.001
Degree of urbanization ^c^	Densely populated area	288	7.3	277	12.2	<0.001
	Intermediate area	367	10.2	271	13.2	<0.001
	Thinly populated area	747	12.1	515	15.4	<0.001
Self- perceived general health	Very good/good	464	5.9	296	6.1	0.292
	Fair (neither good nor bad)	482	13.5	397	21.1	<0.001
	Bad/very bad	456	20.1	370	37.8	<0.001
Chronic disease	No	705	7.6	305	6.1	<0.001
	Yes	697	15.4	758	28.2	<0.001
Limitation in activities due to health problems	Not limited	1008	8.6	700	10.4	<0.001
	Limited	249	18.5	231	35.3	<0.001
	Strongly limited	145	22.0	132	42.3	<0.001
Region ^d^	Serbia-North	565	9.9	419	13.0	<0.001
	Serbia-South	837	10.4	644	14.4	<0.001
Overall		1402	10.2	1063	13.8	<0.001

* Pearson’s chi-squared test; ^a^ ISCED levels grouped in accordance with education system in Serbia: elementary school or less = ISCED 2 or below, high school = ISCED 3, college/universaty = ISCED 4–6, master/doctorate = ISCED 7–8; ^b^ inactive refers to being unable to work due to long-standing health problems or fulfilling domestic tasks or other duties; ^c^ division according to DEGURBA classification: densely populated area—contiguous grid cells of 1 km^2^ with a density of at least 1500 inhabitants per km^2^ and a minimum population of 50,000, intermediate area—clusters of contiguous grid cells of 1 km^2^ with a density of at least 300 inhabitants per km^2^ and a minimum population of 5000, thinly populated area—grid cells outside urban clusters; ^d^ NUTS1-level region Serbia-North includes NUTS2-level regions: Belgrade Region and Region of Vojvodina; NUTS1-level region Serbia-South includes NUTS2-level regions: Region of Sumadija and Western Serbia and Region of Eastern and Southern Serbia.

**Table 3:** Unmet healthcare needs and their predictors- result from multivariate regression analysis.

Variable	Category	Pooled Sample (*N* = 21,422)	
		OR	95% CI
Year	2019	ref.	
	2021	**1.60**	**1.46** **–** **1.75**
Predisposing factors			
Sex	Male	ref.	
	Female	0.94	0.85–1.03
Age group (years)	16–29	ref.	
	30–49	**1.80**	**1.42–2.29**
	50–64	**2.30**	**1.79–2.96**
	65+	**2.32**	**1.74–3.09**
Education level ^a^	Elementary or less	ref.	
	High school	**0.72**	**0.65–0.81**
	College/university	**0.65**	**0.55–0.77**
	Master/doctorate	**0.51**	**0.30–0.88**
Employment status ^b^	Employed	ref.	
	Unemployed	1.05	0.91–1.21
	Retired	**0.70**	**0.58–0.83**
	Inactive	**0.76**	**0.62–0.93**
	Student, pupil	**0.22**	**0.13–0.37**
Enabling factors			
Income quintile	1—poorest	ref.	
	2	**0.77**	**0.68–0.88**
	3	**0.63**	**0.55–0.72**
	4	**0.60**	**0.52–0.70**
	5—richest	**0.49**	**0.41–0.58**
Degree of urbanization ^c^	Densely populated area	ref.	
	Intermediate area	1.13	1.00–1.28
	Thinly populated area	**1.19**	**1.06–1.33**
Health needs			
Self-perceived general health	Very good/good	ref.	
	Fair (neither good nor bad)	**1.85**	**1.63–2.11**
	Bad/very bad	**2.16**	**1.81–2.57**
Chronic disease	No	ref.	
	Yes	**1.** **46**	**1.** **29–1.64**
Geographical classification			
Region ^d^	Serbia-North	ref.	
	Serbia-South	0.98	1.03–1.34

Note: Results are adjusted for marital status and limitations in activities due to health problems. Bold indicates *p* < 0.05; OR  =  odds ratio; CI  =  confidence interval; ref. = reference category; ^a^ ISCED levels grouped in accordance with education system in Serbia: elementary school or less = ISCED 2 or below, high school = ISCED 3, college/universaty = ISCED 4–6, master/doctorate = ISCED 7–8; ^b^ inactive refers to being unable to work due to long-standing health problems or fulfilling domestic tasks or other duties; ^c^ division according to DEGURBA classification: densely populated area—contiguous grid cells of 1 km^2^ with a density of at least 1500 inhabitants per km^2^ and a minimum population of 50,000, intermediate area—clusters of contiguous grid cells of 1 km^2^ with a density of at least 300 inhabitants per km^2^ and a minimum population of 5000, thinly populated area—grid cells outside urban clusters; ^d^ NUTS1-level region Serbia-North includes NUTS2-level regions: Belgrade Region and Region of Vojvodina; NUTS1-level region Serbia-South includes NUTS2-level regions: Region of Sumadija and Western Serbia and Region of Eastern and Southern Serbia.

## Data Availability

The data for this manuscript is covered under the Cooperation Agreement (No. 404-1134 from 13 September 2023), and thus, we are unable to share the datasets regardless of the fact that they are in anonymized form. These restrictions are imposed by the Statistical Office of the Republic of Serbia (https://www.stat.gov.rs/en-US/, accessed on 29 June 2025) and the subsequent Cooperation Agreement between the Faculty of Medicine of University of Belgrade and the Statistical Office of the Republic of Serbia. The data are transferred in accordance with art. 48 of the Law on Official Statistics of the Republic of Serbia (“Official Gazette of RS”, no. 104/2009), which specifically prohibits the submission of the data to third parties.

## Supplementary Materials

The following supporting information can be downloaded at https://www.mdpi.com/article/10.3390/healthcare14050599/s1. Table S1. Factors associated with unmet healthcare needs before and during the COVID-19 pandemic—results from multivariate regression analyses.
