# Supplementary material for: Factors Associated with Unmet Healthcare Needs in Serbia Before and During the COVID-19 Pandemic: Analysis Based on EU-SILC Data
Source: Healthcare (Basel). 2026 Feb 27;14(5):599. doi: 10.3390/healthcare14050599 (PMC12984895; doi:10.3390/healthcare14050599)
Supplement: Supplementary file 1 [file healthcare-14-00599-s001.zip › healthcare-4122762-supplementary.pdf]

Table S1. Factors associated with unmet healthcare needs before and during the COVID-19 pandemic - results from multivariate regression analyses.

Variable	Category	Before COVID-19 (2019) *N*=13733		During COVID-19 (2021) *N*=7689	
		**OR**	**95% CI**	**OR**	**95% CI**
Predisposing factors					
Sex	Male	ref.		ref.	
	Female	**0.86**	**0.76- 0.98**	1.10	0.95- 1.29
Age group (years)	16-29	ref.		ref.	
	30-49	**1.66**	**1.25- 2.19**	**2.45**	**1.52- 3.96**
	50-64	**2.19**	**1.63- 2.95**	**3.25**	**1.99- 5.31**
	65+	**2.17**	**1.53- 3.09**	**3.12**	**1.82- 5.35**
Marital status	Married	ref.		ref.	
	Never married	1.05	0.87- 1.26	1.14	0.89- 1.47
	Separated/divorced	**1.43**	**1.14- 1.80**	**1.73**	**1.28- 2.34**
	Widowed	**1.27**	**1.06- 1.52**	1.14	0.93- 1.38
Education level[a]	Elementary or less	ref.		ref.	
	High school	**0.56**	**0.49- 0.65**	1.05	0.88- 1.26
	College/university	**0.45**	**0.36- 0.58**	1.01	0.78- 1.31
	Master/doctorate	**0.27**	**0.12- 0.62**	1.08	0.52- 2.25
Employment status[b]	Employed	ref.		ref.	
	Unemployed	0.92	0.78- 1.09	1.18	0.92- 1.52
	Retired	**0.44**	**0.35- 0.56**	1.27	0.96- 1.69
	Inactive	**0.54**	**0.41- 0.70**	1.26	0.91- 1.73
	Student, pupil	**0.18**	**0.09- 0.35**	**0.42**	**0.18- 0.95**
Enabling factors					
Income quintile	1- poorest	ref.		ref.	
	2	**0.71**	**0.61- 0.83**	0.91	0.74- 1.12
	3	**0.57**	**0.48- 0.69**	**0.73**	**0.58- 0.91**
	4	**0.56**	**0.46- 0.68**	**0.71**	**0.56 - 0.90**
	5- richest	**0.41**	**0.33- 0.52**	**0.63**	**0.49- 0.82**
Degree of urbanisation[c]	Densely populated area	ref.			ref.
	Intermediate area	**1.25**	**1.06- 1.48**	0.95	0.78- 1.15
	Thinly populated area	**1.20**	**1.03- 1.40**	**1.20**	**1.10- 1.44**
Health needs					
Self-perceived general health	Very good/good	ref.			
	Fair (neither good nor bad)	**1.97**	**1.66- 2.32**	**1.54**	**1.25- 1.89**
	Bad/very bad	**2.38**	**1.90- 2.99**	**1.90**	**1.45- 2.49**
Chronic disease	No	ref.		ref.	
	Yes	1.05	0.90- 1.23	**2.38**	**1.95- 2.89**
Limitation in activities due to health problems	Not limited	ref.		ref.	
	Limited	**1.34**	**1.11- 1.62**	**1.70**	**1.37- 2.11**
	Strongly limited	**1.55**	**1.22- 1.97**	**2.02**	**1.50- 2.72**
Geographical classification					
Region[d]	Serbia -North	ref.		ref.	
	Serbia- South	0.90	0.80- 1.02	1.11	0.95- 1.29

Bold indicates $p<0.05$; OR = odds ratio; CI = confidence interval; ref.=reference category; [a]ISCED levels grouped in accordance with education system in Serbia: elementary school or less = ISCED 2 or below, high school = ISCED 3, college/universaty = ISCED 4-6, master/doctorate = ISCED 7-8; [b] inactive refers to unable to work due to long-standing health problems or fulfilling domestic tasks or other; [c]Division according to DEGURBA classification: densely populated area- contiguous grid cells of 1km^2 with a density of at least

1500 inhabitants per km^2 and a minimum population of 50 000, intermediate area- clusters of contiguous grid cells of $1km^2$ with a density of at least 300 inhabitants per km^2 and a minimum population of 5000, thinly-populated area- grid cells outside urban clusters; [d] NUTS1 level region Serbia-North includes NUTS2 level regions: Belgrade Region and Region of Vojvodina; NUTS1 level region Serbia-South includes NUTS2 level regions: Region of Sumadija and Western Serbia and Region of Eastern and Southern Serbia.
